# Supplementary material for: Nanoscale nonreciprocity via photon-spin-polarized stimulated Raman scattering
Source: Nat Commun. 2019 Jul 24;10:3297. doi: 10.1038/s41467-019-11175-z (PMC6656711; doi:10.1038/s41467-019-11175-z)
Supplement: Supplementary file 1 — Supplementary Information [file 41467_2019_11175_MOESM1_ESM.docx]

**Nanoscale nonreciprocity via photon-spin-polarized stimulated Raman scattering**

**Supplementary Information**

Lawrence *et al.,*

In this supplementary document, we elucidate the specifications of the simulations we have performed and provide further details regarding one-way Raman amplification in silicon metasurfaces.

**Supplementary Note 1: Simulation details**

Throughout the current study, results have been attained by performing frequency domain calculations using the finite element solver COMSOL. Each metasurface design considered in the main text has a lattice period smaller than the shortest illumination wavelength. We thus model transmission through the periodic arrays by applying continuously periodic boundary conditions to the sides of a single unit cell and port boundary conditions in the circularly polarized plane wave basis at the top and bottom of the cell. For Figs. 2-3 of the main text, silicon was used as both the resonator material and the Raman active material, with material parameters given in the text. For Fig. 4, silver was used in the arms of the bowtie with complex permittivity data taken from Johnson and Christy^1^, while diamond was used as the Raman active material with refractive index of 2.4. A Raman shift of 40THz, halfwidth of 60GHz and $\chi_{res}={11.2\times10}^{-18}{(m/V)}^{2}$,^2^ were also used for diamond.

**Supplementary Note 2: Nonreciprocal Raman amplification in silicon metasurfaces**


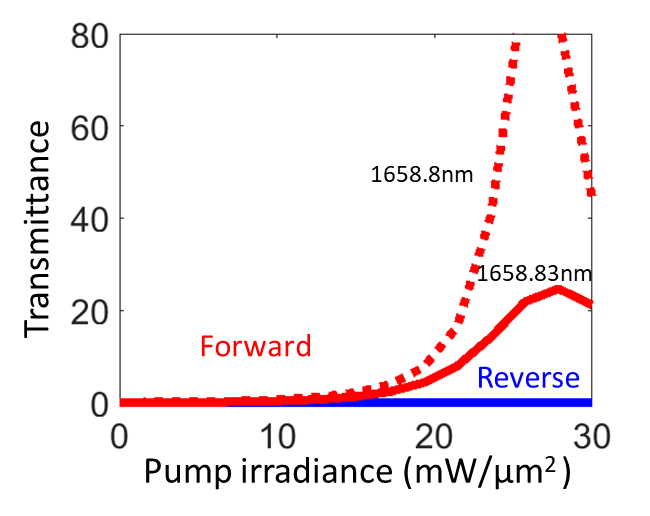
In Fig. 3 of the main text the effect SRS has on the silicon metasurface transmission spectrum for discrete pump powers is shown. To better illustrate the pump power dependence of the device performance, in Supplementary Fig. 1 we plot the continuous pump power spectrum for two discrete probe wavelengths. The wavelengths chosen correspond to the transmission minimum for the unbiased

Supplementary Fig. 1: Directional transmittance as a function of Pump irradiance corresponding to metasurface considered in fig3 of the main text. Probe wavelength is 1658.8nm for dashed curves and 1658.83nm for solid curves.

metasurface, 1658.83nm, and the singular wavelength, 1658.83nm. In both cases we find a very flat response with reverse illumination, blue curves. For forward illumination, red curves, the transmission grows dramatically up until around 27mW/µm^2^, after which it quickly drops again. Comparing the two wavelengths we find much more pronounced amplification at 1658.8nm and 27mW/µm^2^. This wavelength and gain combination actually represents a singularity in the scattering matrix of the structure. If pumped to this level we would expect nonreciprocal lasing to be observed.

**Supplementary Note 3: Double resonant nonreciprocal metasurface**

In the main text, a high Q resonance is used to provide feedback for the low power signal, amplifying the small Raman gain coming from the pump. It is also possible to boost the local gain coefficient for a given

Supplementary Fig. 2: Double resonant nonreciprocal metasurface. a) Transmittance spectrum (black curves) and max field intensities (grey curves) near pump (blue dashed line) and probe (red dashed line) wavelengths. b) Directional SRS induced transmittance of 1534.77nm probe as a function of increasing pump irradiance.

pump illumination by aligning a high Q mode to the pump wavelength. Fig.2b shows that a second high Q mode with magnetic symmetry already exists in the bi-periodic disk array. A much more efficient device can be expected if the frequency separating the two different high Q modes in the system can be tuned to match the Raman phonon energy. To achieve this, we exploit the different trends for the magnetic and electric dipole resonant frequencies with changing aspect ratio of the disks. In Supplementary Fig. 2.a, the Raman side band of the high Q electric resonance of a metasurface made from 220nm tall disks with diameters 604nm and 594nm is seen to fall exactly on the high Q magnetic resonance. More than a 50 fold enhancement of the illuminating electric field strength therefore occurs with this structure at both signal and pump wavelengths. Supplementary Fig. 2.b shows the corresponding directional Raman gain. As a result of the double resonance configuration, enhanced transmission in the forward direction begins to occur for pump powers as low as a few 10s of µW/µm^2^. While the isolation efficiency is slightly diminished compared to the single resonance case, which is due to the poor modal overlap at the center of the disks where the polarisations are most circular, strong nonreciprocity is still produced for such a subwavelength optical device. Improved performance could also be achieved with spectral and modal engineering in more sophisticated structures. The Q factors of the magnetic and electric modes used in Supplementary Fig. 2 are 15000 and 22500, respectively, which are within bounds of experimental measurements on similar systems.^3^

**Supplementary Note 4: Other nonlinear effects**

SRS is a third order optical nonlinearity that scales with the pump light intensity. Other nonlinearities are known to occur in silicon which could interfere with the Raman process when resonantly amplified. To check for this, here we consider two of the most significant nonlinearities, the Kerr effect and two photon absorption (TPA). In Supplementary Fig. 3 we repeat the simulation in Fig. 3c of the main text after applying the Kerr coefficient $\chi^{(3)}=2.79\times{10}^{-18}m^{2}/V^{2}$ within the silicon regions to account for pump induced index shifts, otherwise known as cross-phase modulation.^4^ Strong nonreciprocity is still observed. The transmission is altered in both directions due to a red shift if the electric dipole resonance, but this could be corrected for easily by slightly adjusting the pump wavelength. Next, we include non-degenerate TPA. In this process, the presence of the strong pump field leads to parametric absorption of the probe. In Supplementary Fig. 4, with a large TPA coefficient equivalent to a figure of merit ~0.4,^5^ it can be seen that the SRS enhanced transmission is only slightly effected by TPA in the forward direction, while in the reverse direction the extra absorption actually increases the isolation efficiency.


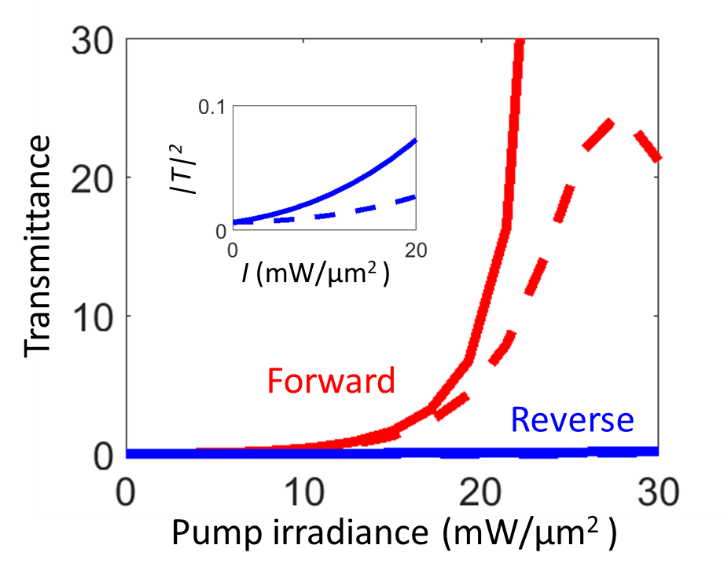


Supplementary Fig. 3: Nonreciprocal transmission through metasurface with SRS and cross phase modulation. Dashed curves include SRS only and solid curves include SRS and Kerr nonlinearity.


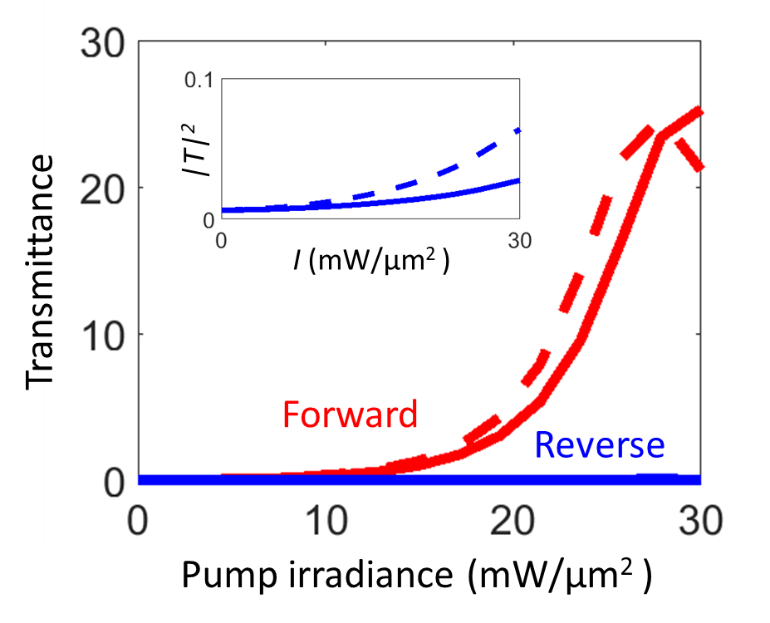


Supplementary Fig. 4: Nonreciprocal transmission through metasurface with SRS and cross amplitude modulation. Dashed curves include SRS only and solid curves include SRS and TPA.

**Supplementary Note 5: Design principles for SRS based nonreciprocal nanophotonics**

A bulk silicon crystal pumped with CPL represents the ideal case for nonreciprocal SRS. A counter-propagating signal is maximally amplified, while a co-propagating signal is entirely unaffected by the pump. A nanostructured device therefore has three roles to play: 1) to boost the efficiency of SRS in the forward direction, 2) suppress transmission in the reverse direction, 3) and maintain suppression of Raman gain in the reverse direction. Our metasurface design achieves 1 and 2 via a high Q resonance and 3 with rotational symmetry. Two figures of merit can be defined using the linear nearfields and equation 6 in the main text which can inform the design of nonreciprocal SRS based devices. The overall SRS efficiency depends on evaluating equation 6 for the forward signal illumination configuration χ­_f_, while the ratio of evaluating equation 6 for forward and reverse configurations χ­_f/_ χ­_r_ relates to the asymmetry in the effective Raman gain coefficient. For our silicon metasurface, χ­_f_ =3.62e9 and χ­_f/_ χ­_r_ =7.42. In Supplementary Fig. 5 we compare the central plane of our silicon metasurface, which well represents the entire mode due to its weak variation in z, and the TE mode of a [100] silicon waveguide. While the waveguide shows large asymmetry χ­_f/_ χ­_r_ =527, the SRS enhancement factor is significantly weaker, with χ­_f_ =2.05e4. As discussed in the main text, the non-zero overlap between co-rotating pump and signal fields, reproduced in Supplementary Fig. 5a, originates from linearly polarized fields. For a waveguide, the transverse and longitudinal fields have a uniform π/2 phase difference and so the pump/signal overlap in the reverse configuration remains small despite the spatial distributions of |Ez| and |Ey| being very different. Comparing Supplementary Fig. 5e and f, however, we see that this cancellation is detrimental to the efficiency of SRS in the forward direction as pump-signal interactions are forbidden at the center of the structure where the E-field is strongest. On the other hand, Supplementary Fig. 5c and d are almost identical, showing that pump-signal interactions are near optimal in the metasurface.

Finally, we note that the waveguide only shows nonreciprocal amplification and does not suppress transmission in the reverse direction. The strong asymmetry of the waveguide also relies on the [100] crystal orientation. The field overlap in Supplementary Fig. 5d, and thus reverse Raman gain, quickly grows as the crystal is rotated towards [110]. Much weaker nonreciprocity should therefore be expected upon constructing a ring cavity from such a waveguide. While χ­_f_ and χ­_f/_ χ­_r_  are important factors for designing and improving the performance of nonreciprocal Raman based devices, ultimately, the suitability of such a system within a given application must be determined by the scattering parameters and from Figs. 3b and c we can clearly see that our subwavelength metasurface exhibits a strong nonreciprocal response.


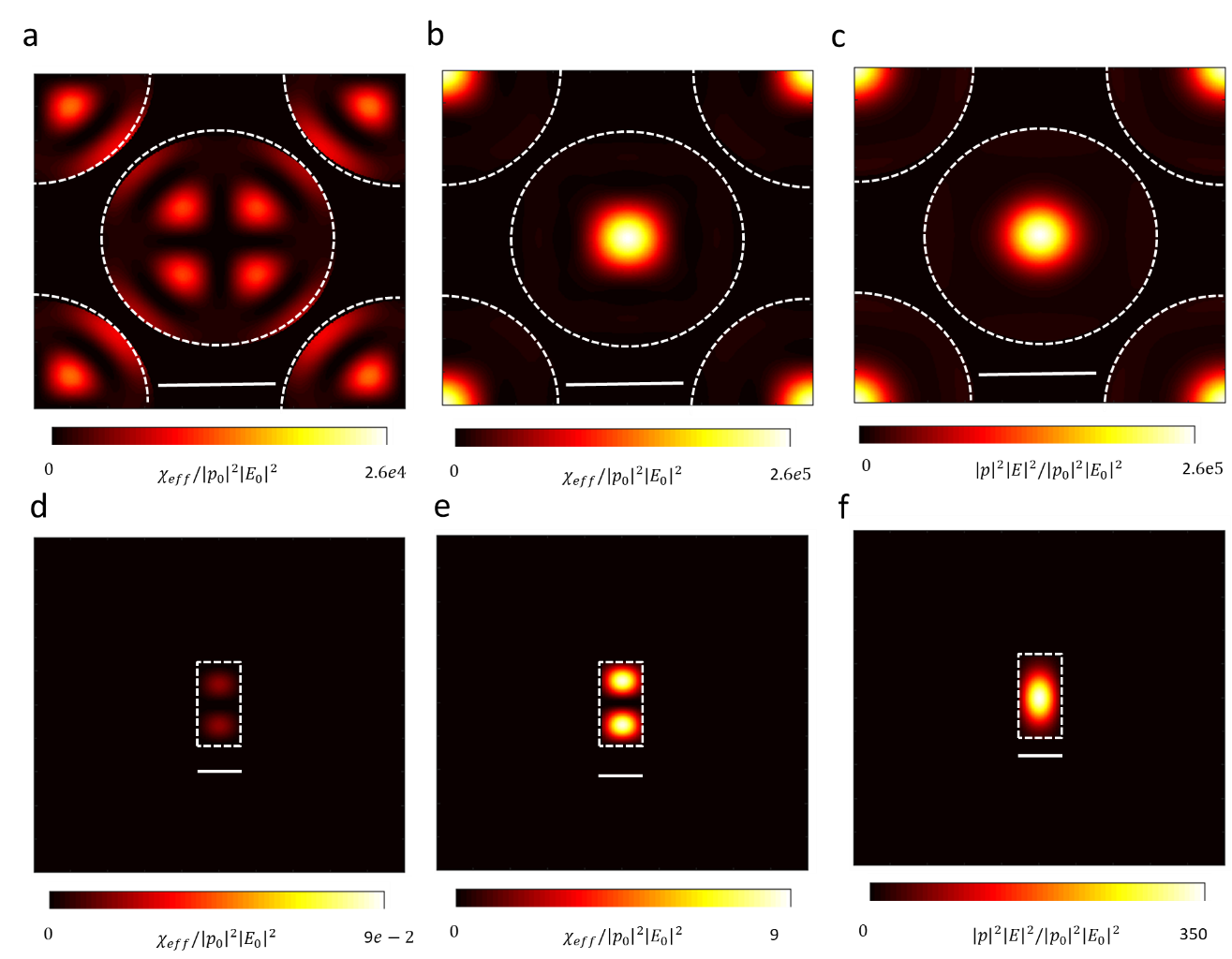


Supplementary Fig. 5: Effective Raman susceptibility for silicon metasurface (a-c) and silicon waveguide (d-f). Raman susceptibility, i.e. pump and probe overlap with Raman tensor, in reverse direction (a and d), and forward direction (b and e). Norm squared product of pump and probe fields (c and f). scale bar is 325 nm (a-c) and 220 nm (d-f).

**Supplementary Note 6: Designing a circular polarizer**

In the main text we focus on breaking time reversal symmetry. For circularly polarized light, helicity is conserved under time reversal. So, the time reverse of a left handed wave is still left handed but travelling in the opposite direction. Waves with opposite helicity are instead related by inversion symmetry. Supplementary Fig. 6 shows the power dependent response of our inversion symmetric metasurface to right handed CPL. Strong nonreciprocity with almost identical power dependence is seen except for the exchange of forward and reverse directions.

Supplementary Fig. 6: Directional transmittance as a function of Pump irradiance corresponding to metasurface considered in Fig. 3 of the main text, but with right handed signal.


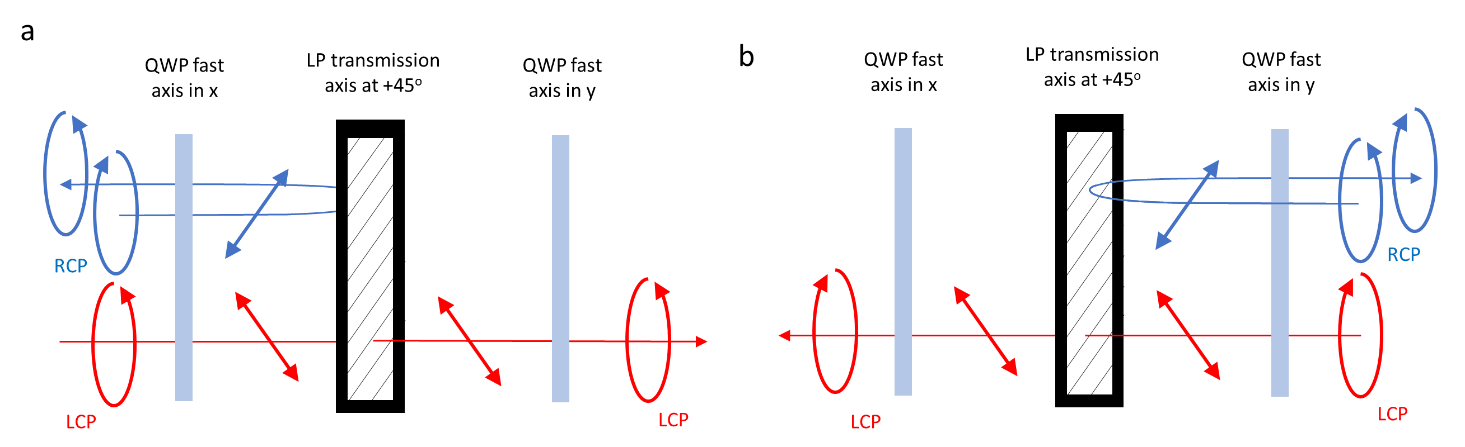
Waves with opposite helicity can be distinguished by arranging passive dielectric materials asymmetrically in space. Supplementary Fig. 7 shows schematically how this can be achieved with a simple stack of traditional optical

Supplementary Fig. 7: Circular polarizer constructed from linear polarizer sandwiched between two quarter waveplates. a) left incidence and b) right incidence.

components. The stack consists of a linear polarizer sandwiched between two quarter waveplates. By rotating one quarter waveplate by 90 degrees with respect to the other, the incident helicity will be preserved upon passive through. But in-between the waveplates left and right handed incident waves are transformed into orthogonal linear polarisations. The linear polarizer is then able to select a particular handedness to be transmitted while the other is reflected. Comparing Supplementary Fig. 7a and Supplementary Fig. 7b, we can see that Left handed light is transmitted in both directions while right handed light is reflected with helicity preserved. Placing our nonreciprocal spin selective metasurface in front of this device would produce full optical isolation. This device could also be shrunk down to the nanoscale using resonant chiral nanoantennas, or even incorporated into our nonreciprocal scheme by inducing stimulated Raman scattering in a chiral metasurface.

**Supplementary References**

(1) Johnson, P. B.; Christy, R. W. *Phys. Rev. B* **1972**, *6* (12), 4370–4379.

(2) Okawachi, Y.; Yu, M.; Venkataraman, V.; Latawiec, P. M.; Griffith, A. G.; Lipson, M.; Lončar, M.; Gaeta, A. L. *Opt. Lett.* **2017**, *42* (14), 2786.

(3) Liu, Y.; Wang, S.; Zhao, D.; Zhou, W.; Sun, Y. *Opt. Express* **2017**, *25* (9), 10536.

(4) Yang, Y.; Wang, W.; Boulesbaa, A.; Kravchenko, I. I.; Briggs, D. P.; Puretzky, A.; Geohegan, D.; Valentine, J. *Nano Lett.* **2015**, *15* (11), 7388–7393.

(5) Leuthold, J.; Koos, C.; Freude, W. *Nat. Photonics* **2010**, *4* (8), 535–544.
